# Case Report: Neuroblastoma Breakpoint Family Genes Associate With 1q21 Copy Number Variation Disorders

**DOI:** 10.3389/fgene.2021.728816

**Published:** 2021-09-27

**Authors:** Lijuan Zhu, Xiaoji Su

**Affiliations:** Children's Hospital of Fudan University Anhui Hospital, Hefei, China

**Keywords:** 1q21.2, microduplication, hearing loss, congenital heart disease, Olduvai domain

## Abstract

Microduplications and reciprocal microdeletions of chromosome 1q21. 1 and/or 1q21.2 have been linked to variable clinical features, but the underlying pathogenic gene(s) remain unclear. Here we report that distinct microduplications were detected on chromosome 1q21.2 (GRCh37/hg19) in a mother (255 kb in size) and her newborn daughter (443 kb in size), while the same paternal locus was wild-type. Although the two microduplications largely overlap in genomic sequence (183 kb overlapping), the mother showed no clinical phenotype while the daughter presented with several features that are commonly observed on 1q21 microduplication or microdeletion patients, including developmental delay, craniofacial dysmorphism, congenital heart disease and sensorineural hearing loss. *NBPF15* and *NBPF16*, two involved genes that are exclusively duplicated in the proband, may be the cause of the clinical manifestations. This study supports an association between *NBPF* genes and 1q21 copy number variation disorders.

## Introduction

Extensive DNA repeats on chromosome 1q21.1 and 1q21.2 predispose the regions to recurrent rearrangement through non-allelic homologous recombination, which generates microduplication and microdeletion events. Despite variable penetrance, affected patients tend to present with developmental delays (Cooper et al., 2011), congenital heart disease (Christiansen et al., 2004; Mefford et al., 2008), intellectual disability (Sharp et al., 2006), macrocephaly/microcephaly (Brunetti-Pierri et al., 2008), autism spectrum disorder (Dolcetti et al., 2013), schizophrenia (International Schizophrenia Consortium, 2008; Stefansson et al., 2008), as well as neuroblastoma (Diskin et al., 2009). Hearing impairment is also commonly noticed on patients bearing either 1q21 microduplication (Rosenfeld et al., 2012; Dolcetti et al., 2013; Bernier et al., 2016; Pang et al., 2020) or the microdeletion (Brunetti-Pierri et al., 2008; Harvard et al., 2011; Rosenfeld et al., 2012), however this phenotype/genotype association has not been investigated specifically.

Since sizes of the 1q21 microduplications and reciprocal microdeletions can range from hundreds of kilobases to megabase, and dozens of genes may be involved, it is difficult to establish an association of a clinical feature with a specific gene. The *GJA5* gene, encoding Cx40, has been linked to cardiac malformation in 1q21.1 microdeletion and microduplication carriers (Christiansen et al., 2004; Soemedi et al., 2012; Guida et al., 2013). Also, a role for Cx40 in heart development had also been validated in genetic knockout mice (Gu et al., 2003). Regarding the macrocephaly/microcephaly feature, the *HYDIN2* gene, the paralog of *HYDIN*, was believed to be the cause of the abnormality (Brunetti-Pierri et al., 2008). However, genetic involvement in hearing impairment remains unclear in 1q21 copy number variation carriers. Here, we report a case observation where a newborn infant bearing 1q21.2 microduplication presents with mild developmental delays, facial dysmorphism, atrial septal defect and sensorineural hearing loss, with only two *NBPF* genes involved.

## Results

**Figure 1:**
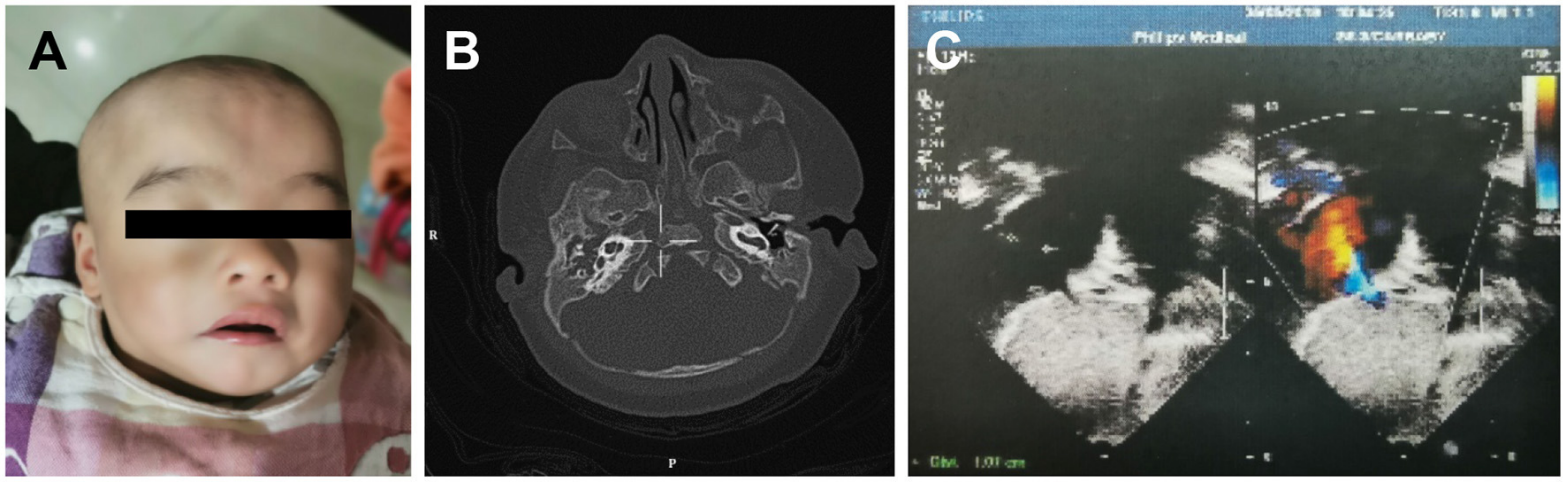
The developmental anomalies of the proband. **(A)** Patient shows facial dysmorphism. **(B)** Computerized tomography scan shows tympanosclerosis and blockage of the ear canals. **(C)** Echocardiogram showing a 1.1 cm atrial septal defect.

A 3-month-old female infant, presenting with mild developmental delay and facial dysmorphism (Figure 1A), was admitted due to a failed newborn hearing screen. To confirm the patient's hearing capabilities, an auditory steady-state response (ASSR) and auditory brainstem response (ABR) tests were performed. The ASSR thresholds for the patient's left ear were measured as 80, 80, and 70 dB at carrier frequencies of 500, 1,000, and 2,000 Hz, respectively. The thresholds for the right ear were all 100 dB at the same measured frequencies. Consistently, in the ABR test, evoked potentials were observed for the left ear, but not the right ear, at 80 and 90 dB stimuli. A computed tomography (CT) scan showed that both ears, but primarily the right ear, presented with tympanosclerosis, in addition to a blockage of the right ear canal (Figure 1B). In addition to the hearing deficit, an echocardiogram revealed a 1.1 cm atrial septal defect (ASD) and hypertrophy of right atrium and right ventricle, indicating congenital heart disease (Figure 1C).

Based on the observations of these unrelated developmental defects, a gene mutation was suspected as the cause of the malformations. Mutations of mitochondrial DNA have been linked to developmental abnormalities such as deafness and congenital heart disease (Taylor and Turnbull, 2005). We thus sequenced mitochondrial DNA of peripheral blood cells (PBCs) from both affected proband and her non-affected mother, by means of long range PCR and next generation sequencing (Cui et al., 2013) with a depth coverage of 50,000x. However, the 3 identified mutations (m.6962G>A on *COX1* gene, m.7447A>G on *TRSN1* gene and m.16129G>A on Dloop_e region) were all maternally inherited, making them unlikely candidates for the proband's developmental abnormalities (Table 1).

**Table 1:** The three mitochondrial DNA mutations identified in proband and her mother.

**Gene/position**	**Mutation**	**Amino acid change**	**Mutation rate in proband (%)**	**Mutation rate in mother (%)**
*COX1*	m.6962G>A	Leu>Leu	99.91	99.85
*TRNS1, tRNA*	m.7447A>G	NA	99.54	99.52
*Dloop_e*	m.16129G>A	NA	99.87	99.86

To further investigate the involved pathogenesis, whole exome sequencing was performed using PBCs derived from the proband as well as her non-affected parents. Those point mutations in the proband with allele frequency below 0.2 or allele depth below 4 are regarded as unreliable and excluded. The remaining variants are then classified, according to ACMG practice guidelines (Richards et al., 2015) and OMIM database (https://www.omim.org/), as benign or potentially pathogenic. Finally, 11 missense point mutations of 8 proteins in the proband that could be potentially related to the developmental disorders were identified (Table 2). However, none of these mutations were likely to be the cause of the congenital anomalies, as they are all heterozygous and inherited from either of the non-affected parents who are also heterozygotes.

**Table 2:** Identified missense mutations in proband and their status in her parents.

**Gene**	**Site(s)**	**Chromosome**	**Mutation**	**Amino acid change**	**Patient**	**Father**	**Mother**
*ADAMTSL2*	1	chr9: 136419545	c.1006(exon10)G>A	p.E336K (NM_001145320)	Hetero.	Hetero.	Wild type
*FAT4*	1	chr4: 126370225	c.8054(exon9)G>A	p.R2685Q (NM_024582)	Hetero.	Hetero.	Wild type
*FMN2*	1	chr1: 240371881	c.3769(exon5)C>G	p.P1257A (NM_020066)	Hetero.	Hetero.	Wild type
*MCM4*	1	chr8: 48877210	c.770(exon7)A>G	p.Q257R (NM_005914)	Hetero.	Hetero.	Wild type
	2	chr8: 48883929	c.1829(exon12)G>A	p.R610H (NM_005914)	Hetero.	Wild type	Hetero.
*PIEZO1*	1	chr16: 88783550	c.6541(exon45)C>T	p.L2181F (NM_001142864)	Hetero.	Wild type	Hetero.
	2	chr16: 88798228	c.3082(exon22)C>T	p.R1028C (NM_001142864)	Hetero.	Hetero.	Wild type
*POLG*	1	chr15: 89865008	c.2557(exon16)C>T	p.R853W (NM_002693)	Hetero.	Wild type	Hetero.
*SFTPC*	1	chr8: 22021459	c.481(exon5)C>T	p.R161X, 31 (NM_001172357)	Hetero.	Hetero.	Wild type
*SHROOM4*	1	chrX: 50377042	c.2031(exon4)T>G	p.S677S (NM_020717)	Hetero.	Hetero.	Wild type
	2	chrX: 50351006	c.3136(exon6)C>T	p.L1046F (NM_020717)	Hetero.	Wild type	Hetero.

**Figure 2:**
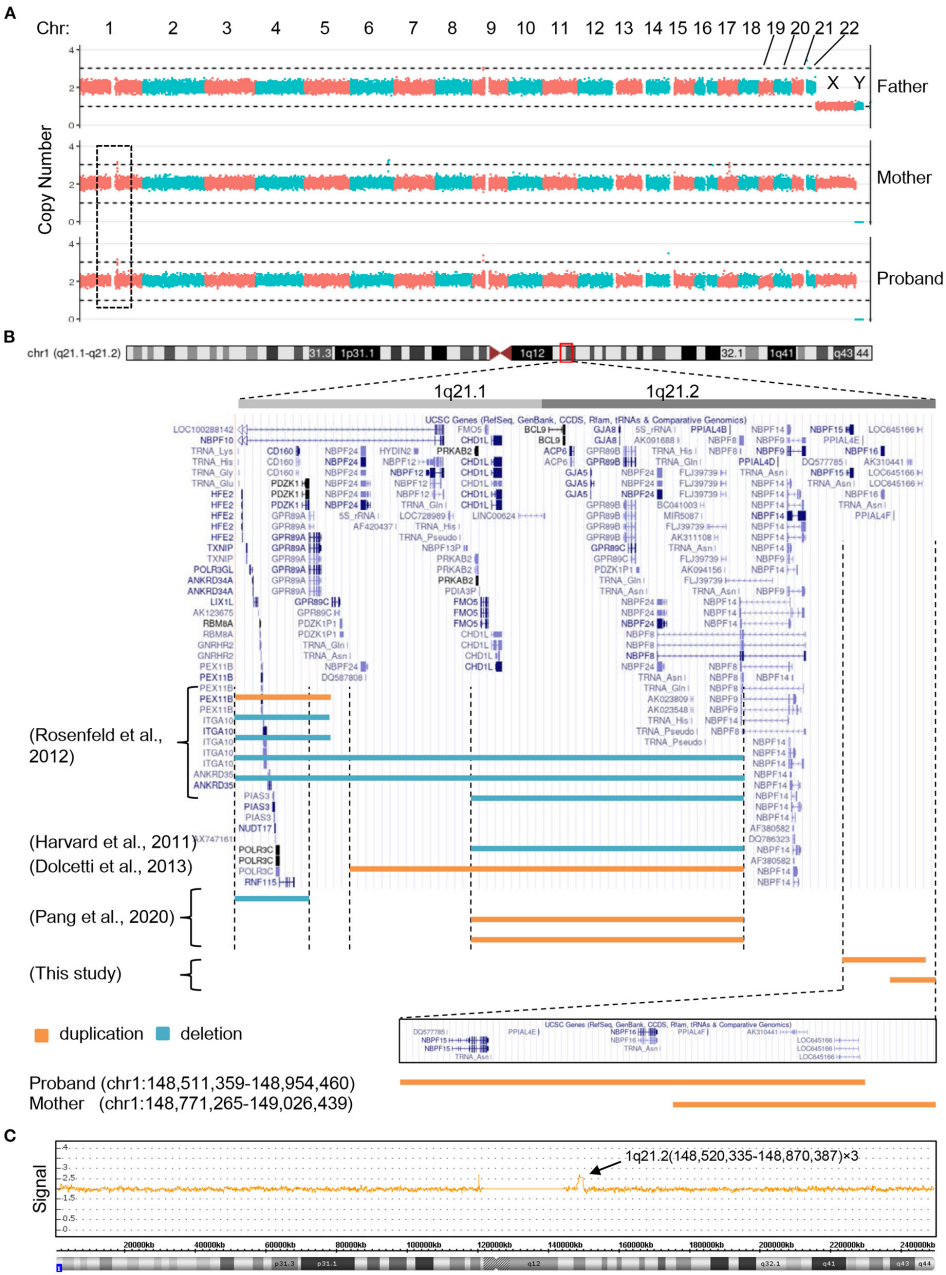
The 1q21 microduplications or microdeletions of the proband, her mother and other reported patients with hearing problem. **(A)** Copy number analysis of the proband and her parents by next generation sequencing. **(B)** Positional plots of the microduplications and microdeletions on 1q21.1 and 1q21.2 regions (http://genome.ucsc.edu). **(C)** Confirmation of the proband's microduplication by CytoScan HD array.

Having ruled out the possibilities that mutations of mitochondrial DNA or genomic coding sequence were the cause of the clinical manifestations, we next evaluated if copy number changes apply to the patient's genomic DNA. To this end, whole genome sequencing of PBCs derived from the proband and her parents was performed and the copy number variation was analyzed. A microduplication (copy number = 3) on chromosome 1 was observed in the proband and maternal genomic DNA, but was lacking from the paternal genome (Figure 2A). The microduplication in the proband (chr1:148,511,359–148,954,460) was distinct from that of her mother (chr1:148,771,265–149,026,439), although both duplications arose on chromosome 1q21.2 and the duplicated fragments largely overlapped (Figure 2B). Chromosome microarray of the proband's genomic DNA confirmed the 1q21.2 microduplication (Figure 2C). *NBPF15* and *NBPF16* are the only involved genes specific to the proband, suggesting that these two genes might be the causative agents of the congenital abnormalities. Consistent with this observation, and expanding upon it, *NBPF* genes are repeatedly found to be involved in the 1q21 microduplication or microdeletion in patients with congenital hearing deficits (Figure 2B).

## Discussion

Although this study only involves one patient, it is unique in several ways; first, the microduplicated segment of the proband is 1q21.2 specific, contrary to the majority of reported 1q21 copy number changes, which primarily occur in the 1q21.1 region (Brunetti-Pierri et al., 2008; Mefford et al., 2008; Diskin et al., 2009; Harvard et al., 2011; Rosenfeld et al., 2012; Soemedi et al., 2012; Dolcetti et al., 2013; Bernier et al., 2016; Pang et al., 2020). Despite this difference, the proband shares similar clinical features with 1q21.1 microduplication or microdeletion carriers, such as developmental delays, craniofacial dysmorphism, congenital heart disease and hearing loss. The second unique feature of this study is that the proband's microduplication is distinct from that of her mother. While both microduplications are largely overlap, the difference seen in the proband and her mother may be indicative of *de novo* 1q21.2 rearrangements. Last but not the least, there are only two genes from the same gene family (*NBPF15* and *NBPF16*) specifically involved in the proband's 1q21.2 microduplication, which strongly suggests the genetic pathogenesis.

*NBPF* genes contain multiple copies of DUF1220/Olduvai domain which has been linked to human brain evolution (Popesco et al., 2006; O'Bleness et al., 2012). Of the ~300 haploid copies of human Olduvai domain, about 80% localize in 1q21.1 and 1q21.2 chromosomal regions; the driver of Olduvai domain expansion is thought to remain active in the general population (Heft et al., 2020), which is consistent with patients' recurrent rearrangement of the 1q21 region. Clinically, an increase in Olduvai copy number shows a linear association with severity of autism symptoms (Davis et al., 2014, 2015, 2019), while a decrease in the copy number is associated with schizophrenia (Searles Quick et al., 2015). These reported observations are in line with the associations of 1q21 microduplications and microdeletions with autism and schizophrenia, respectively (International Schizophrenia Consortium, 2008; Stefansson et al., 2008; Dolcetti et al., 2013). Olduvai copy number has also been proven to be linearly associated with brain size (Dumas et al., 2012), which justifies the macrocephaly/microcephaly feature in 1q21 copy number variation carriers.

In conclusion, our case observation supports an association between *NBPF* genes and 1q21 microduplication and microdeletion syndromes. However, it does not rule out the potential role of other genes involved in the observed pathogenesis.

## Data Availability Statement

The datasets for this article are not publicly available due to concerns regarding participant/patient anonymity. Requests to access the datasets should be directed to the corresponding author.

## Ethics Statement

The studies involving human participants were reviewed and approved by Ethics Committee of Children's Hospital of Fudan University Anhui Hospital. Written informed consent to participate in this study was provided by the participants' legal guardian/next of kin. Written informed consent was obtained from the minor(s)' legal guardian/next of kin for the publication of any potentially identifiable images or data included in this article.

## Author Contributions

LZ and XS conducted the research. LZ analyzed data and drafted the manuscript. Both authors contributed to the article and approved the submitted version.

## Funding

This study was supported by the Hospital's Internal Research fund to LZ.

## Conflict of Interest

The authors declare that the research was conducted in the absence of any commercial or financial relationships that could be construed as a potential conflict of interest.

## Publisher's Note

All claims expressed in this article are solely those of the authors and do not necessarily represent those of their affiliated organizations, or those of the publisher, the editors and the reviewers. Any product that may be evaluated in this article, or claim that may be made by its manufacturer, is not guaranteed or endorsed by the publisher.
